# Free Radicals as a Double-Edged Sword: The Cancer Preventive and Therapeutic Roles of Curcumin

**DOI:** 10.3390/molecules25225390

**Published:** 2020-11-18

**Authors:** Nehal Gupta, Kshitij Verma, Sarath Nalla, Alok Kulshreshtha, Rajiv Lall, Sahdeo Prasad

**Affiliations:** 1 Department of Medicine, University of California San Francisco, 1600 Divisadero Street, San Francisco, CA 94115, USA; nehal.gupta@ucsf.edu; 2 Discovery Chemistry, Genentech, Inc., 1 DNA Way, South San Francisco, CA 94080, USA; verma.kshitij@gene.com; 3 Noble Pharma, LLC, 4602 Domain Drive, Menomonie, WI 54751, USA; sarathn@noblepharmallc.com (S.N.); Alokk@noblepharmallc.com (A.K.); lallr123@yahoo.com (R.L.)

**Keywords:** free radicals, ROS, cancer, curcumin, chemoprevention, therapy

## Abstract

Free radicals, generally composed of reactive oxygen species (ROS) and reactive nitrogen species (RNS), are generated in the body by various endogenous and exogenous systems. The overproduction of free radicals is known to cause several chronic diseases including cancer. However, increased production of free radicals by chemotherapeutic drugs is also associated with apoptosis in cancer cells, indicating the dual nature of free radicals. Among various natural compounds, curcumin manifests as an antioxidant in normal cells that helps in the prevention of carcinogenesis. It also acts as a prooxidant in cancer cells and is associated with inducing apoptosis. Curcumin quenches free radicals, induces antioxidant enzymes (catalase, superoxide dismutase, glutathione peroxidase), and upregulates antioxidative protein markers–Nrf2 and HO-1 that lead to the suppression of cellular oxidative stress. In cancer cells, curcumin aggressively increases ROS that results in DNA damage and subsequently cancer cell death. It also sensitizes drug-resistant cancer cells and increases the anticancer effects of chemotherapeutic drugs. Thus, curcumin shows beneficial effects in prevention, treatment and chemosensitization of cancer cells. In this review, we will discuss the dual role of free radicals as well as the chemopreventive and chemotherapeutic effects of curcumin and its analogues against cancer.

## 1. Introduction

Free radicals are generally reactive oxygen species (ROS) and reactive nitrogen species (RNS), which are capable of independent existence. ROS and RNS can broadly be categorized into two groups—radicals and non-radicals. Superoxide (O_2_^−•^), oxygen radical (O_2_^••^), hydroxyl (OH^•^), alkoxyradical (RO^•^), peroxyl radical (ROO^••^), nitric oxide (NO^•^) and nitrogen dioxide (NO_2_^•^) are various examples of radical species [1]. The non-radical species are comprised of hydrogen peroxide (H_2_O_2_), hypochlorous acid (HOCl), hypobromous acid (HOBr), ozone (O_3_), singlet oxygen (^1^O_2_), nitrous acid (HNO_2_), nitrosyl cation (NO^+^), nitroxyl anion (NO^−^), dinitrogen trioxide (N_2_O_3_), dinitrogen tetraoxide (N_2_O_4_), nitronium cation (NO_2_^+^), organic peroxides (ROOH), aldehydes (HCOR) and peroxynitrite (ONOOH). Radical species are comparatively highly reactive, due to the presence of an unpaired electron that imparts a high degree of electrophilicity. These free radicals act as oxidants or reductants by either donating an electron to or accepting an electron from other reactive molecules [2].

Free radicals are generated in the body by various endogenous and exogenous systems including pathological states and exposure to different physiochemical conditions. The formation of free radicals in the body is a continuous process through the enzymatic and non-enzymatic reactions. Mitochondria, peroxisomes, and phagocytic organelles participate in the enzymatic production of free radicals while non-enzymatic free radicals are produced by ionizing radiation and non-enzymatic reactions of oxygen with organic compounds [3]. Accumulated evidences suggest that free radicals cause progressive adverse effects in the body by increasing oxidative stress. Although the body has an antioxidant defense mechanism to balance the redox system, excessive production of ROS and RNS results in oxidative and nitrosative stress respectively. This chronic oxidative or nitrosative stress manifests in the form of a number of diseases including cancer. This is evident by a notion that the overproduction of free radicals has a close relation with increased incidences of cancer. It has also been found that a higher consumption of fats and oils leads to the increased production of free radicals correlated with the increased death rate from different types of cancer [4].

To combat the detrimental effects of free radical species ROS and RNS, a system of antioxidant defense is expressed in all living cells. The antioxidant system can be classified in multiple ways. It may be based on the source as exogenous (derived from dietary food intake) or endogenous (produced in the body), solubility in water (e.g., vitamin C) or lipids (e.g., vitamin E), or the size and nature of the antioxidant molecule, i.e., enzymatic (e.g., catalase) or non-enzymatic (e.g., flavonoids). Studies have shown that antioxidants reduce the occurrence of carcinogenesis. It has been observed that the consumption of fruits and vegetables, rich in antioxidants, in daily diet causes a reduction in the risk for cancer incidence [5]. In contrast, several studies have also shown that antioxidant from dietary supplements such as β-carotene and retinol promote tumor growth and metastasis in cancer patients [6,7,8]. However, various anticancer drugs kill cancer cells by producing free radicals [9]. Thus, these studies indicate that free radicals serve a dual purpose—inducing carcinogenesis and imparting in cancer cell death.

## 2. Free Radicals as a Double Edge Sword

Reactive oxygen species (ROS), produced either exogenously (e.g., radiation, chemicals, hyperoxia) or endogenously (normal cellular metabolism), are related to a wide variety of human disorders. Excessive ROS causes damage to proteins, DNA, and RNA leading to genetic alterations in cells. On the other hand, low levels of ROS are essential for various biological functions, including cell survival, cell growth, proliferation and differentiation, and immune response [10]. Lethal production of ROS by certain agents also causes death of cancer cells. These observations indicate the dual role of ROS in terms of cancer promoting and cancer suppressing activity. It is evident that ROS participates simultaneously in both the Ras-Raf-MEK1/2-ERK1/2 oncogenic signaling and the p38 mitogen-activated protein kinases (p38MAPK) tumor suppressing pathways [11]. Thus, depending on the cell type, ROS may function as cytoprotective or oncogenic.

### 2.1. Carcinogenesis

The high accumulation of ROS and/or low level of antioxidants in cells causes an imbalance in redox status, which is known as oxidative stress. Oxidative stress is implicated in a variety of pathologies including cancer. ROS are short-lived and highly electrophilic molecules generated by the partial reduction of oxygen to form superoxide (O_2_^−•^), hydrogen peroxide (H_2_O_2_), and hydroxyl radical (HO^•^) as well as secondary metabolites including lipid peroxides, peroxynitrite (ONOO^−^), and hypochlorous acid (HOCl). Because of their high reactivity, they cause damage to the macromolecules and disrupt biochemical function [12].

It has been known that ROS causes DNA strand breaks and oxidative damage to the nucleotides, subsequently resulting in mutagenesis and eventually cancer. The susceptible target of ROS in DNA is guanine that causes G→T transversions [13]. Additionally, ROS can also cause mutations by oxidative damage to a range of target sites in genetic materials including purines and pyrimidines, alkali labile sites, single strand breaks and disruption of DNA repair processes, leading to genetic instability [14,15]. ROS-induced carcinogenesis is supported by a study describing that elevated levels of ROS leads to modification of nucleobases in cancerous and precancerous tissues [16]. The initiation of cancer in humans caused by ROS is further supported by the presence of oxidative DNA modifications in cancer tissues [17]. Oxidative DNA damage leading to the development of breast cancer has also been reported. For instance, in inflammatory breast cancer, an increase in DNA base damage and 8-oxo-dG adducts leads to malignant cancer progression [18]. The role of oxidative stress in the development of hepatocellular carcinoma has also been reported, since ROS caused accumulation of 8-OHdG by oxidative DNA damage in the cells and further development of hepatocellular carcinoma [19]. The association of oxidative DNA damage and carcinogenesis have been found in a variety of other cancers. However, a comparative measurement of distinctively modified DNA bases in tumor tissue and their respective normal tissues is required to provide further insights into the involvement of ROS in carcinogenesis.

ROS also induces modifications of redox-sensitive amino acid residues in regulatory proteins including cysteine oxidation. Such modifications can modulate the regulatory effects of proteins and enzymes. The regulatory proteins such as kinases (MAPK and PI3K/Akt), transcription factors (Nrf2, AP-1, NF-κB, STAT3, and p53), components of ubiquitin/proteasome system and autophagy/lysosomal protein degradation systems, molecular chaperones, and cytoskeletal proteins are susceptible to altered physiological function by ROS [20]. These modifications in the regulatory proteins of non-cancerous cells dysregulate cellular homeostasis and as a consequence lead to initiation of carcinogenesis. Stimulation of these signaling pathways by ROS is also found to be involved in proliferation, migration, and invasion of human breast, liver, prostate, lung, skin, pancreatic, and many other cancer cells. Lipids are the other cellular target of ROS. ROS reacts with polyunsaturated or polydesaturated fatty acids to initiate lipid peroxidation [21]. The peroxidation of lipids generate numerous genotoxic molecules such as malondialdehyde, 2-alkenals and 4-hydroxy-2-alkenals [22]. ROS-induced lipid peroxidation may result in alteration of membrane structure, disruption in membrane permeability and interruption in immune system recognition, which cumulatively promotes cancer initiation and progression. The involvement of lipid peroxidation in carcinogenesis was made evident by the presence of thiobarbituric acid-reactive substances (a byproduct of lipid peroxidation) in the serum of patients with colorectal cancer [23].

### 2.2. Therapeutic

Cancer cells have a hypermetabolism that produces a large amount of ROS compared to normal cells. At the same time, cancer cells have marked antioxidant capacity that help in maintaining the redox balance. Recently, anticancer therapies that induce oxidative stress by increasing ROS and/or inhibiting antioxidant level have received great attention [24]. The increased ROS disrupts redox homeostasis and causes damage to the cancer cells and ultimately cell death. Numerous anticancer drugs utilize the principle of oxidative stress-induced chemotherapy.

Since cancer cells possess comparatively high ROS levels, these cells have a higher sensitivity towards increased prooxidant and decreased antioxidant levels [25,26]. This increased sensitivity to prooxidants induces cancer cell death using the process of apoptosis, necroptosis, and autophagy [27], which mediates either by direct induction of ROS generation and/or suppression of antioxidant levels [28]. Some anticancer drugs induce ROS generation and cause cancer cell death. For example, motexafin gadolinium, an electron acceptor, suppresses the irradiation-induced DNA repair and increases the effect of radiotherapy in many cancers [29,30,31]. Anthracycline-based anticancer drugs—such as doxorubicin—also accumulate hydroxyl radicals and cause the death of cancer cells [32]. Another drug, 2-methoxyestradiol, induces mitochondrial production of hydrogen peroxide [33] and subsequently activates c-Jun N-terminal kinase (JNK), resulting in initiation of apoptosis [34,35]; additionally, it can also potentiate the therapeutic action of other anticancer agents [36,37]

Besides these, several natural compounds also exhibit anticancer effects by inducing ROS. Resveratrol, a dietary product present in grapes, vegetables and berries, has been shown to exert an anti-cancer effects on melanoma cells through the induction of ROS. It has also been shown to induce ROS-p38-p53 pathway by increasing the gene expression of phosphorylated p38 MAPK and activating the p53 and ER stress pathway [38]. Ursolic acid, another natural compound, has been shown to induce apoptosis and enhance oxaliplatin-induced inhibition of colorectal cancer cell proliferation in both in vitro and in xenograft nude mouse models. The enhanced anti-proliferative effect of ursolic acid was found to be correlated with increased ROS production and decreased expression of drug resistance genes [39]. A combination of gambogic acid, a natural compound derived from the gamboge, (*Garcinia* species), also caused synergistic reduction of cell viability in SKOV-3 cells with doxorubicin and this effect was found to be correlated with increased cellular ROS accumulation [40]. Similarly, there are several other bioactive dietary polyphenols including quercetin, curcumin, capsaicin, epigallocatechin-3-gallate, piperine, phenethyl isothiocyanate, benzyl isothiocyanate, and others that exert antitumor effects by inducing ROS-mediated cytotoxicity in cancer cells [41].

Other types of anticancer drugs cause cancer cell death by depleting antioxidant levels. For instance, buthionine sulfoximine synergistically affects the chlorin e6 (Ce6)-based photodynamic therapy (PDT) of colorectal cancer cells [42] and several other cancer types including prostate, breast, lung, colon, cervix, bladder, and kidney cancers by inhibiting glutathione [43]. Similarly, imexon—another small-molecule chemotherapeutic agent—disrupts glutathione activity and induces cancer cell death. This agent is widely used in the treatment of advanced cancers of the breast, lung, and prostate [44,45]. Although such anticancer drugs disrupt the redox homeostasis in cancer cells, the inhibition of antioxidant enzymes may also introduce the liability of causing deleterious side effects in normal tissues and organs.

## 3. Cancer Preventive Role of Curcumin through Suppression of Free Radicals

**Figure 1:**
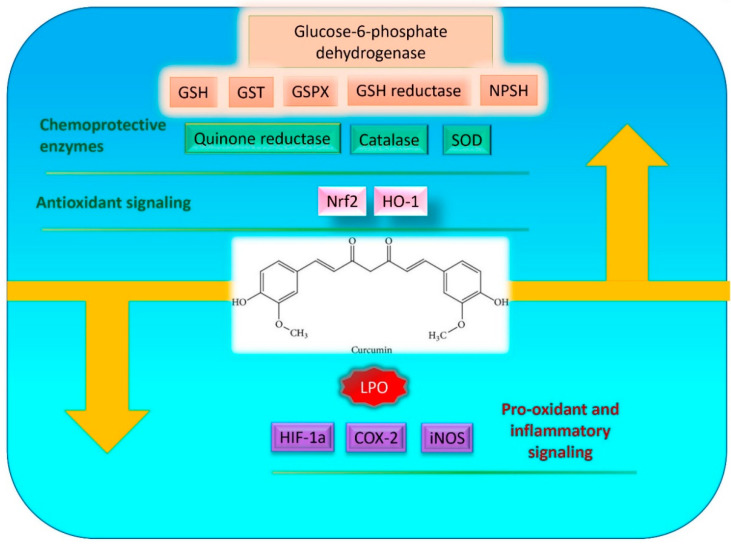
Chemopreventive role of curcumin by suppressing reactive oxygen species (ROS).

As described above, ROS plays a key role in carcinogenesis. Therefore, reducing ROS levels can reserve the initiation of tumorigenesis. Curcumin has shown to be a well-established antioxidant compound by innumerable researchers. Various studies have also shown that curcumin blocks the tumorigenesis process in many rodent carcinogenesis models including skin, duodenal, forestomach, and colon carcinogenesis and therefore works as a chemopreventive agent [46,47,48]. It regulates multiple cell signaling pathways including oxidative stress and inhibits the initiation of carcinogenesis (Figure 1). The chemopreventive effect of curcumin was found to be enhanced when combined with other chemopreventive agents.

### 3.1. Curcumin Mediates Chemopreventive Effect through Its Antioxidant Property

As curcumin has antioxidant activity, it exhibits a chemopreventive effect by reducing ROS levels and by inducing antioxidant enzymes (Table 1). In a study, curcumin has shown chemopreventive property by suppressing colon carcinogenesis in azoxymethane-dextran sulfate sodium (AOM-DSS) treated mice. The chemopreventive effect of curcumin was found to be associated with the downregulation of multiple regulatory pathways including oxidative stress, as analyzed by RNA sequencing of tumors [49]. In another study, curcumin was found to prevent amino-1-methyl-6-phenylimidazo [4,5-b]pyridine (PhIP)-induced cytotoxicity in normal breast epithelial cells. It has been shown that curcumin prevents PhIP-induced DNA adduct formation and DNA double stand breaks through induction of various antioxidant enzyme systems [50]. Curcumin has also been shown to have protective effect against lung carcinogenesis through its antioxidant property. Curcumin at a dose of 60 mg/kg in drinking water suppressed benzopyrene induced ROS and lipid peroxidation in mice. Moreover, curcumin restored benzopyrene-induced reduced level of glutathione in mice, indicating the protective role of curcumin as an antioxidant [51]. Curcumin also inhibited methylglyoxal (MG)-induced apoptotic events in human hepatoma G2 cells. It has been found that curcumin abolishes MG-stimulated intracellular ROS formation, and subsequent apoptotic biochemical changes in hepatoma G2 cells [52].

Curcumin also exhibits potential benefit against arsenic-induced oxidative stress in humans. A field trial was conducted in volunteers consuming arsenic contaminated ground water. Among 286 volunteers, half were treated with curcumin (500 mg twice daily) and remaining half were assigned with placebo. After three months, DNA damage and oxidative stress were analyzed in blood. The blood samples from endemic regions showed severe DNA damage with increased levels of ROS and lipid peroxidation. The antioxidant enzymes showed depleted activity in the placebo treated group. However, curcumin intervention reduced the DNA damage, retarded ROS generation and lipid peroxidation and raised the level of antioxidant activity [53]. Thus, curcumin exhibits a protective role in humans.

Curcumin also exerts chemopreventive efficacy by increasing the activity of antioxidant enzymes and phase II-metabolic enzymes in the liver and kidneys. Curcumin (2%) when fed to mice for 30 days showed a significant enhancement in the activities of glutathione peroxidase, glutathione reductase, glucose-6-phosphate dehydrogenase, and catalase. In addition, curcumin enhances the activities of glutathione *S*-transferase and quinone reductase. This suggests that curcumin may prevent liver and kidney carcinogenesis by inducing antioxidant enzymes [57]. Moreover, in an in vivo rat model, curcumin decreased the oxidative stress levels and showed a dose dependent increase in the NPSH levels in rat liver, which corresponds roughly to tissue glutathione content. Glutathione peroxidase activity was also found to be elevated with increasing curcumin dosing [58], while it reduced lipid peroxidation and salvaged hepatic glutathione antioxidant defense in rats [65]. Such observations clearly point towards the chemopreventive actions of curcumin by inhibiting ROS and inducing antioxidant activities.

The transcription factor Nrf2 is believed to be one of the crucial players in regulating the various antioxidant genes including hemeoxygenase (HO-1). Activation of the Nrf2 antioxidant pathway signaling is reported to play an important role in cancer prevention [66]. Interestingly, curcumin has been shown to activate Nrf2 and HO-1 signaling. In one study, curcumin activated Nrf2 by promoting its nuclear translocation in a time-dependent manner in neuronal cells, and also induced HO-1 protein levels in a concentration-dependent manner [62]. Similarly, curcumin treatment also promoted translocation of Nrf2 protein into the nucleus in renal epithelial cells. This translocation was associated with elevated HO-1 activity [59]. In addition, curcumin treatment increased the expression of HO-1 in vascular endothelial cells leading to the alleviation of oxidative damage [60]. It has also been shown that dietary curcumin consumption at a dose of 53mg/day reduced ovarian cancer incidence in hens. Curcumin was mixed with a basal diet and were mixed homogenously. This curcumin mixed diet was administered to hens. Interestingly, this chemopreventive effect of curcumin was found to be mediated through reduction in oxidative stress and lipid peroxidation in serum and ovarian tissues. The mechanism behind the chemopreventive effect of curcumin in hen ovarian tissue was an upregulation of antioxidant related proteins such as Nrf2 and HO-1 [61]. Another transcription factor, hypoxia-inducible factor 1 (HIF-1) is known to be associated with increased ROS levels in hypoxic conditions [67,68]. In vitro studies have indicated that curcumin can inhibit the expression of HIF-1 in hepatic carcinoma cells [55,69], possibly suggesting the inhibition of hypoxia-induced ROS by curcumin.

Curcumin is a known potent anti-inflammatory agent that prevents tumor progression and exerts a chemopreventive effect on carcinogenesis via suppressing inflammation. Increasing evidences suggest the involvement of ROS as a mechanism by which chronic inflammation drives cancer initiation and progression. Chronic inflammation stimulates ROS generation which leads to tissue injury and promotes carcinogenesis [70,71,72]. TNFα is an inflammatory cytokine that activates NF-κB and increases expression of other inflammatory enzymes such as COX-2 and inducible nitric oxide synthase (iNOS). Importantly, studies support that a positive feedback loop between TNFα and ROS promote inflammation-induced carcinogenesis [73,74,75,76]. Curcumin has shown to prevent colon carcinogenesis in mice and cholangiocarcinogenesis in hamsters by suppressing the expression of pro-inflammatory enzymes iNOS and COX-2 [77,78]. Curcumin also reduced the iNOS mRNA expression in ex vivo cultured macrophages in a concentration-dependent manner [79]. Moreover, it inhibits thioacetamide (TAA)-induced liver inflammation and fibrosis in rats. It further reduced oxidative stress in liver, inhibited apoptosis, and induced autophagy, and thus protects the first dysplastic stage of hepatocellular carcinoma (HCC) in rats [63]. These studies suggest the potential benefit of curcumin as a chemopreventive agent by suppressing inflammation induced ROS.

Considering the poor absorption and bioavailability of curcumin, several analogues of curcumin have been synthesized and optimized for better antioxidant properties [80]. Dimethoxy curcumin, when tested in human peripheral blood mononuclear cells showed a decrease in lipid peroxidation status and an increase in the catalase activity [81]. In another study, efforts have been directed towards synthesizing curcumin derivatives as Nrf2 activators. Curcumin nanoparticles as polylactide co-glycolide (PLGA) nanocapsulated curcumin have shown chemopreventive activity against diethylnitrosamine (DEN) induced HCC in rat. Oral administration of curcumin nanoparticles (20 mg/kg body weight for 16 weeks) in DEN induced HCC rats exerted a significant protection against HCC by restoring redox homeostasis in liver cells [56]. In order to enhance solubility of curcumin, it is encapsulated with biodegradable nanoparticulate formulation based on PLGA and a stabilizer polyethylene glycol (PEG)-5000. In line with the previous studies, the synthesized compounds were found to be cytoprotective against oxidative stress [82].

### 3.2. Chemopreventive Potential of Curcumin in Combination with Other Compounds

The chemopreventive efficacy of curcumin has been evaluated in combination with several other natural and synthetic compounds. Interestingly, the chemopreventive potential of curcumin in combination with resveratrol has been shown in multiple studies. Curcumin in combination with resveratrol prevented lung carcinogenesis in rats [83]. In a study, supplementation with curcumin and resveratrol to benzo(a)pyrene-treated mice resulted in the diminution of the molecular events during the promotional phase of lung carcinogenesis. Curcumin (60 mg/kg orally in drinking water) and resveratrol (5.7 µg/mL orally in drinking water) were administered individually or in combination—3 times a week for a total duration of 22 weeks—to benzo(a)pyrene-treated mice. Supplementation of curcumin and resveratrol alone resulted in improvement in cellular integrity, nuclear deformation and premature mitochondrial senescence, however the effects were potentiated with the combined supplementation of phytochemicals [84]. Benzo(a)pyrene treatment also caused significant increase in the levels of lipid peroxidation along with reduction in glutathione levels and the decreased activities of SOD in lungs of mice. Administration of curcumin and resveratrol to benzo(a)pyrene-treated mice decreased the levels of lipid peroxidation and elevated the activities of SOD respectively. Further, the combination also showed an increase in the levels of both reduced glutathione and SOD activity along with decreased level of lipid peroxidation when compared with the monotherapy [85]. Another study showed that treatment of resveratrol and curcumin in combination significantly decreased the benzo(a)pyrene-induced lipid peroxidation, and restored several antioxidant enzymes including SOD, GR, and GST, which eventually may have contributed to lung cancer prevention [54]. Interestingly, curcumin has shown to reduce doxorubicin-induced cardiotoxicity by inhibiting production of ROS. Additionally, curcumin has synergistic effects with doxorubicin against MCF-7 breast cancer cells [64]. Thus, use of curcumin as an adjuvant, alongside other chemopreventive agents could have substantial clinical implications to further develop novel modalities for chemoprevention.

## 4. Cancer Therapeutic Role of Curcumin through Induction of ROS

**Figure 2:**
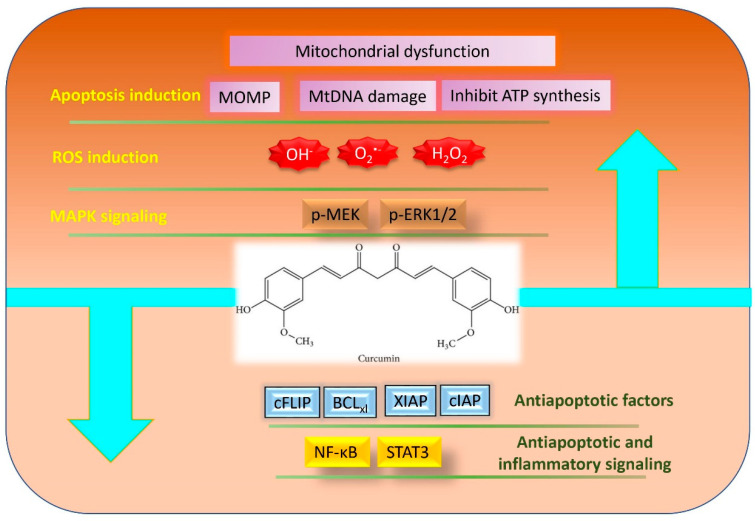
Chemotherapeutic role of curcumin mediated through induction of ROS.

The cancer therapeutic benefits of curcumin have been demonstrated in multiple types of cancers including, breast, prostate, skin, lung, liver, brain, stomach, cervical, ovary, multiple myeloma, leukemia and several other types of cancer [86]. Prior experimental evidences have shown that curcumin is effective in potentiating and enhancing anticancer effects of other cancer therapeutic drugs. Moreover, it sensitizes the drug resistant cells and increases therapeutic potential of anticancer drugs [87]. At the molecular level, curcumin exerts anticancer and chemosensitizing effects by targeting multiple pathways including ROS mediated cell signaling (Figure 2).

### 4.1. ROS Mediated Anticancer Effect of Curcumin

Along with chemopreventive properties, curcumin has also chemotherapeutic effects against different types of cancers through multiple mechanisms including oxidative stress pathway (Table 2). Curcumin regulates cellular redox balance by disrupting mitochondrial homeostasis and enhancing cellular oxidative stress. It oxidizes thiols in the mitochondrial membrane, triggers opening of mitochondrial permeability transition pore, mitochondrial swelling, loss of mitochondrial membrane potential, and inhibition of ATP synthesis, leading to initiation of cellular apoptosis in cancer cells [88]. Curcumin further increases the generation of variety of ROS, including hydroxy radicals, superoxides, and H_2_O_2_ [89,90]. Indeed, in gastric cancer cells curcumin induced the generation of excessive ROS that resulted in depletion of mtDNA and POLG (polymerase γ that reduce mitochondrial oxygen consumption) and subsequent occurrence of cell death [91]. Furthermore, curcumin induces ROS production and decreases mitochondrial transmembrane potential thereby activating DNA damage/repair pathway and mitochondrial apoptosis. Similarly, curcumin exhibited cytotoxicity in NSCLC through the accumulation of cellular ROS. The involvement of ROS was confirmed by using ROS scavengers like catalase and N-acetyl-l-cysteine (NAC) that reversed curcumin-induced cell death in NSCLC [92].

It has also been observed that the treatment of leukemic cells with curcumin increases intracellular ROS levels over a threshold which was found to be linked with the induction of anti-tumorigenic activity [108]. In melanoma A375 cells, curcumin-induced cell death was also found to be associated with increased oxidative stress. It has been shown that curcumin causes oxidative stress through inducing ROS burst, decreasing glutathione, and wrecking mitochondria membrane potential (MMP), which were reversed by ROS inhibitor NAC [94]. Curcumin also affects the signaling pathways regulated by ROS. In human gastric cancer BGC-823 cells, curcumin induced apoptosis through the generation of ROS as evidenced by the inhibition of curcumin-mediated apoptosis upon antioxidant (NAC or trion) application. Notably, it has been observed that curcumin activated oxidative stress-related kinase ASK1, up-regulated an upstream effector of JNK, MKK4, and phosphorylated JNK protein expression in BGC-823 cells [95]. The modulation of these proteins by curcumin resulted in apoptosis in BGC-823 cells. Similarly, in colon cancer cells, curcumin treatment markedly decreased its cell viability and proliferation potential through generation of ROS. However, the pretreatment with NAC suppressed the growth inhibitory effect of curcumin on HT-29 cells [99].

Along with ROS production, depletion of antioxidant enzymes enhances the anticancer property of curcumin. In a study, curcumin suppressed the growth of human leukemic cells via ROS-independent glutathione depletion, which leads to caspase activation and further apoptosis in leukemic cells. Curcumin treatment to leukemic cells also downregulates the expression of the inhibitor of apoptosis proteins (IAPs), phospho-Akt, c-Myc, and cyclin D1, probably through the suppression of glutathione [96]. In another study, curcumin induced apoptosis in MCF-7, MDAMB-231, and HepG2 cells through generation of ROS. However, depletion of glutathione by buthionine sulfoximine resulted in increased generation of ROS and that enhanced curcumin-mediated apoptosis [97]. It has been reported that curcumin causes rapid generation of ROS in human cutaneous T-cell lymphoma (HuT-78) cells. This increased ROS further modulated different cell survival and cell death pathways and subsequently induced apoptosis. Curcumin also downregulates the expression of antiapoptotic proteins c-FLIP, Bcl-xL, cellular inhibitor of apoptosis protein (cIAP), and X-linked IAP (XIAP) in a ROS-dependent manner, which further induces cancer cell death [109]. Besides apoptosis, curcumin also induces autophagic cell death in HCT116 human colon cancer cell by inducing ROS production [110]. Curcumin also hinders cancer stemness through production of ROS. It inhibits proliferation, sphere forming and colony forming abilities of glioblastoma [111] and liver cancer [112] stem cells by ROS mediated activation of MAPK pathway, suppression of NF-κB signaling, downregulation of STAT3 activity and IAP family members.

**Figure 3:**
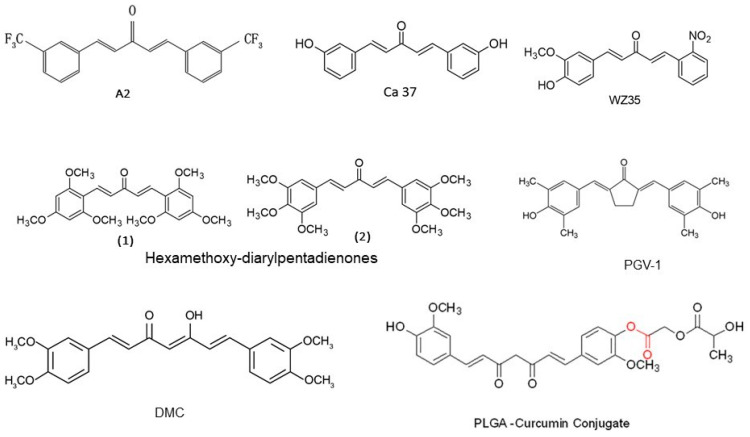
Chemical structure of curcumin analogues, which exhibits anticancer effects mediated through ROS.

Besides curcumin, its analogues (Figure 3) also exhibit ROS mediated chemotherapeutic activity against various cancers. In a study, curcumin analogue 1,5-bis(3-hydroxyphenyl)-1,4-pentadiene-3-one (Ca 37), which is a monocarbonyl analogue of curcumin, exhibited antitumor activity in both in vitro and in prostate xenografted tumor models. Furthermore, a combination of Ca 37 and curcumin resulted in enhanced antitumor activity in prostate cancer cells. However, administration of ROS quencher NAC abrogated Ca 37 mediated tumor growth inhibition; indicating that induction of ROS plays a vital role in the growth inhibitory activity of Ca 37 in prostate cancer cells [101]. In another study, two symmetrical hexamethoxy-diarylpentadienones (1 and 2) monocarbonyl analogues of curcumin have been reported to possess significantly enhanced cytotoxicity as compared to the parent molecule. In comparison to curcumin, these analogues induce a more potent burst of ROS [103]. WZ35, another structural monocarbonyl analogue of curcumin, was examined for anti-prostate cancer effects in both in vitro and in vivo models and showed a reduction in cancer cell viability, an increase in apoptosis, and G2/M cell cycle arrest through overproduction of ROS. Interestingly, WZ35-induced apoptosis in prostate cancer cells was found to be completely reversed by ROS inhibition. Additionally, in an animal study, WZ35 inhibited prostate homograft tumor growth with increased ROS accumulation, mitochondrial disruption, and cell apoptosis in tumor tissues [102], indicating ROS plays a key role in mediating WZ35 tumor growth inhibition.

Another monocarbonyl analogue of curcumin, **A2** without the β-diketone moiety, displayed antiangiogenic activity. It has been demonstrated that **A2** exerts its antiangiogenic activity mainly through inducing endothelial cell death via elevating NADH/NADPH oxidase-derived ROS [106]. Furthermore, curcumin analogue pentagamavunon-1 (**PGV**-**1**) has been studied for its inhibitory activity on tumor cells in vitro and in vivo. **PGV**-**1** (2,5-bis-(4-hydroxy, 3′,5′-dimethyl)-benzylidine-cyclopentanone) is a monocarbonyl analogue of curcumin and has stronger potency than the lead compound, curcumin. It has been found that **PGV**-**1** exhibits 60-fold higher efficacy compared to that of curcumin in inhibiting K562 cell growth. **PGV**-**1** also inhibited the proliferation of leukemia, breast adenocarcinoma, cervical cancer, uterine cancer, and pancreatic cancer cells. Interestingly, **PGV**-**1**-induced cell death is mediated by an increase in intracellular ROS levels through inhibition of ROS-metabolic enzymes [100]. Nakamae et al. (2019) synthesized 39 novel curcumin derivatives and examined their anti-proliferative and anti-tumorigenic properties. They found that these analogues exhibit anti-proliferative activity toward human cancer cell lines in a manner sensitive to antioxidant NAC. Some analogues markedly increased ROS levels and efficiently induced cell death as well as suppressed tumor formation in a xenograft mouse model. Finally, it was found that the anti-tumorigenic activity of these analogues was well-correlated with an increase in ROS levels [105]. Furthermore, nanoformulation of curcumin (curcumin-loaded nanoemulsion) increased intracellular curcumin accumulation and ROS formation, while preventing migration and invasion of melanoma cells [107].

### 4.2. Chemosensitizing Effect of Curcumin Mediated through ROS

Accumulated preclinical evidences suggest that the effectiveness of chemotherapy is being limited due to drug resistance, therapeutic selectivity, and undesirable side effects. In this regard, curcumin has proved its ability to sensitize drug-resistant cancer cells and enhance therapeutic efficacy of drugs. As ROS production by curcumin plays a pivotal role in cancer cell death, it can also mediate chemosensitization. Studies showed that curcumin improves the sensitivity of cancer cells to chemotherapy drugs by regulating a variety of signaling pathways [113]. An in vitro study demonstrated that cisplatin in combination with curcumin exhibit higher anti-tumor activity in HepG2 cells compared with mono-drug therapy. This synergistic effect was found to be the result of increased intracellular ROS levels in HCC cells [114]. Another study showed that curcumin also exhibits a pro-oxidant effect and exerts a chemosensitive property. It sensitizes cisplatin-resistant cells by targeting Nrf-2, NF-κB and STAT3 phosphorylation [115]. The apoptosis in bladder cancer (253J-Bv and T24) cells was found to be increased when curcumin was treated in combination with cisplatin as compared to single agent treatment. The synergistic induction of apoptosis by curcumin and cisplatin was associated with increased ROS production together with upregulation of p-MEK and p-ERK1/2 signaling [116].

The cancer therapeutic response of curcumin has been shown to be improved by tolfenamic acid (a non-steroidal anti-inflammatory drug). Tolfenamic acid has been reported to inhibit the growth of human cancer cells in vitro and in vivo. However, the combination of tolfenamic acid and curcumin showed higher growth inhibition when compared to either single agent. Further, another study showed that the combination treatment upregulated the ROS level that led to an increase in apoptosis in colorectal cancer cells [117]. Subtoxic concentrations of curcumin has also demonstrated to sensitize human renal cancer cells to the tumor necrosis factor-related apoptosis inducing ligand (TRAIL)-mediated apoptosis. Curcumin induces generation of ROS that led to the expression of death receptor 5 (DR5) and further enhancement in TRAIL mediated apoptosis [118]. An increase in ROS production and mitochondria depolarization by the combined treatment of curcumin and tamoxifen was also observed. This combination of drugs resulted in synergistic induction of autophagy along with apoptosis in chemoresistant melanoma cells, indicating the role of curcumin-induced ROS in combination chemotherapy [119]. ROS generation by curcumin has also been shown to enhance irinotecan’s therapeutic effects on colorectal cancer cells by inhibiting cell viability and inducing cell cycle arrest and apoptosis [120]. Curcumin analogue also sensitizes drug-resistant cancer cells. In a study, curcumin analog ALZ003 inhibited the survival of TMZ-sensitive and -resistant glioblastoma in both in vitro and in vivo models through the accumulation of ROS, lipid peroxidation and suppression of GPX4 [121]. Demethoxycurcumin (DMC), a naturally occurring curcumin analogue has been shown to enhance antitumor effect of temozolomide (TMZ). In a study, treatment of DMC prior to TMZ in glioblastoma cells resulted in a significant increase in caspase-3 signaling, mitochondria-related apoptosis and a marked inhibition of cell growth in vitro through production of ROS as well as inactivation of JAK/STAT3 signaling pathway [104].

Combinations of drugs may enhance the therapeutic effect of individual drug treatment. Combinations of natural products are broadly explored in cancer therapy. In a study, curcumin was combined with arabinogalactan (found in Larch trees) and treated to the human breast cancer cells. This combination promoted cell growth inhibition and apoptosis induction in human breast cancer cells by increasing ROS level as well as loss of mitochondrial membrane potential and reduction of glutathione. In addition, this combination inhibited the progression of breast tumors in mouse model [122]. It has been observed that curcumin in combination with quercetin induced apoptosis by increasing ROS and decreasing glutathione levels, as well as by inducing loss of mitochondrial membrane potential. The combination of quercetin and curcumin potentiates individual’s apoptotic effect and reduces effective dose of individual agent [93]. Nanoparticles loaded with curcumin and quercetin have also shown synergistic antitumor properties on a breast cancer cell line. Cell viability study showed a pronounced antitumor effect in combination compared to the individual drug on the MCF7 cell line, which was associated with an increase in intracellular ROS level [123]. Curcumin and resveratrol have also been found to exhibit a synergistic anticancer effect in colon cancer. The combination of curcumin and resveratrol has shown to upregulate intracellular ROS levels and further elicit a synergistic antiproliferative effect in Hepa1-6 cells [124]. Besides these, there are several natural compounds as well as therapeutic drugs that have shown to exhibit synergistic anticancer effect with curcumin mediated by ROS induction.

## 5. Conclusions

Oxidative stress—caused by an imbalance between free radical generation and the antioxidant defense system of the body—potentially results in the initiation and progression of cancer. However, free radicals, specially ROS, play an important role in therapeutic drug action. Thus, ROS has both pathological and pharmacological importance in the body. These findings suggest the use of strategies to either suppress or induce ROS. One of the strategies is to decrease oxidative stress by quenching ROS and/or increasing antioxidant enzymes that can subsequently inhibit carcinogenesis. Another strategy is to use ROS inducing agents in cancer patients that can induce apoptosis in cancer cells. Use of antioxidants in cancer patients have shown unfavorable results for the treatment efficacy of therapeutic drugs. Studies have shown that antioxidants cause increased cancer incidences, probably due to the inhibition of ROS mediated apoptosis of cancer cells. However, high consumption of fruits and vegetables rich in antioxidants are associated with decreased risk of cancer incidence. In view of this, curcumin is considered as an antioxidant as well as a prooxidant, since it reduces oxidative stress in normal cells and induces ROS production in cancer cells. Although several studies have investigated the beneficial role of curcumin, this review will help to better understand the ROS associated chemopreventive and anticancer mechanism of curcumin.

## Figures and Tables

**Table 1:** Cancer preventive properties of curcumin mediated through inhibition of oxidative stress.

Properties	Models	Mechanisms	References
Anti-carcinogenesis	BP-induced lung tumor in mice	Decreases the levels of LPO, ROS, as well as increases activities of SOD, GST	[51]
		In combination with resveratrol, decreases the LPO level and restores activities of SOD, GR, and GST	[54]
	CoCl2-induced hypoxia in HCC	decreased hypoxia-induced HIF-1α protein, suppressed cell proliferation, migration and invasiveness, as well as EMT changes	[55]
	AOM-DSS-induced colon cancer in mice	Decreases DNA CpG methylation of Tnf	[49]
	DEN induced HCC in rats	Combats oxidative damage of hepatic cells and inhibits carcinogenesis	[56]
Chemopreventive	ddY mice	Increases the activity of antioxidant enzymes GPx, GR, glucose-6-phosphate dehydrogenase and catalase	[57]
	Sprague-Dawley rats.	Increases activity of GST enzyme	[58]
	Renal epithelial cells	Stimulates the expression of Nrf2, increases in HO-1	[59]
	Bovine aortic endothelial cells	Increases the expression of HO-1 mRNA, protein and its activity	[60]
	Spontaneous ovarian cancer in hen	Reduces tumor sizes and number, inhibits NF-κB and STAT3 signaling pathways, decreases KRAS and HRAS mutations, and induces NRF2/HO-1 antioxidant pathway	[61]
Chemoprotective	Hemin-induced cytotoxicity in rat neurons.	Attenuates ROS production, reduces GSH/GSSG ratio, increases GR, GST and SOD enzymes, increases HO-1 level and Nrf2 translocation into the nucleus, and reduces cell death	[62]
	TAA-induced liver inflammation and fibrosis in rats	Reduces oxidative stress, inhibits apoptosis, induced autophagy, decreases fetoprotein AST activity, and increased serum albumin concentration.	[63]
Anti-cytotoxic	PhIP-induced cytotoxicity in breast epithelial cells	Decreases ROS production, inhibits DNA adduct formation and DNA double stand breaks, and induces expression of various antioxidant and DNA repair genes	[50]
	Dox-induced cytotoxicity in 3T3 normal cells	With resveratrol and EEAC increases cell antioxidant ability by improving the activity of SOD, prevents intracellular damage, and reduces ROS	[64]
	MG-induced cell death in human hepatoma G2 cells	Abolishes oxidative stress, prevents apoptotic biochemical changes such as release of cytochrome c, caspase-3 activation, and cleavage of PARP	[52]

TAA—Thioacetamide, AST—Aspartate aminotransferase, MG-Methylglyoxal, EEAC—Ethanolic extract of Antrodia cinnamomea, SOD—Superoxide dismutase, ROS—Reactive oxygen species, PhIP-Amino-1-methyl-6-phenylimidazo [4,5-b]pyridine, Nrf2—nuclear factor erythroid 2–related factor 2, HO-1—heme oxygenase-1, GSH—glutathione, GSSG—glutathione disulfide, GR—glutathione reductase, GPx—glutathione peroxidase, GST—glutathione-S-transferase, LPO—lipid peroxidation, AOM—azoxymethane-dextran sulfate.

**Table 2:** Cancer therapeutic properties of curcumin and its analogues mediated through generation.

Properties	Models	Mechanism	Reference
Apoptosis	Myeloid leukemia K562 cells	Releases cytochrome c from mitochondria, PARP and caspase-9 cleavages	[93]
	Melanoma A375 cells	Induces ROS burst, decreases GSH, and wrecks MMP	[94]
	Gastric cancer BGC-823 cells	Induces ROS, activates ASK1, and phosphorylates JNK protein	[95]
	Leukemic Jurkat and K562 cells	Downregulates IAPs, pAkt, c-Myc, and cyclin D1	[96]
	Breast cancer MCF-7, MDAMB, HepG2 cells	Generates ROS	[97]
Cell cycle arrest	Breast cancer MCF-7 cells	Downregulates cyclin B1, Cdc2 and NF-κB by decreasing the interaction of pIκB-NF-κB	[98]
Cell cycle arrest and apoptosis	HT-29 colon cancer cells	Induced ROS generation, DNA fragmentation, chromatin condensation, and nuclear shrinkage	[99]
	K562 cells and xenograft mouse	Derivative **PGV-1** induces prometaphase arrest in the M phase and induces cell senescence and death by increasing ROS.	[100]
	Prostate carcinoma PC-3 and DU145 and xenograft mice	Analogue Ca 37 induces ROS production	[101]
	Prostate cancer RM-1 and DU145 cell lines and xenograft mice	Analog WZ35 induces ROS overproduction, intracellular calcium surge, and mitochondrial disruption	[102]
	NCI-H460 cells	Analogues hexamethoxy-diarylpentadienones (1 and 2) upregulate p53 and p21 and downregulate Cdc25C, cyclin B1/Cdk1 in a Michael acceptor- and ROS-dependent fashion	[103]
	NSCLC A549 and SPC-A1 cell lines	Causes ROS production, DNA damage, endoplasmic reticulum stress and mitochondrial instability.	[92]
Chemosensitization	Glioblastoma	DMC synergistically increases TMZ-induced apoptosis by increasing ROS production, inactivating JAK/STAT3 signaling pathway and caspase-3 cleavage	[104]
Anti-tumorigenesis	CML-derived K562 cells, xenograft mouse	Derivatives upregulate ROS levels, compete with co-enzymes to bind to the respective ROS metabolic enzymes and inhibit their activities	[105]
Anti-angiogenesis	HUVECs, CAMs	Analog **A2** induces NADH/NADPH oxidase-derived ROS	[106]
Tumor re-incidence and metastasis inhibition	B16F10 cells, syngeneic mice	Nanoformulation increases intracellular curcumin accumulation and ROS formation	[107]
Anti-tumorigenesis	Gastric cancer BGC-823 cells, xenograft mice	Enhances oxidative stress, decreases mtDNA content and DNA polymerase γ	[91]
	Leukemic K562 cells, xenograft mice	Induces ROS level	[108]
Autophagy and apoptosis	lymphoma HuT-78 cells	Produces ROS, inhibits c-FLIP, Bcl-xL, cIAP, XIAP, disrupts the integrity of IKK and beclin-1 by degrading Hsp90, inhibits NF-κB, accumulates autophagy marker LC3-I	[109]
Autophagy	Colon cancer HCT116 cells	Generates ROS, converts autophagic marker LC3-I to LC3-II and degrades sequestome-1	[110]

MMP—matrix metallopeptidase, ASK1—apoptosis signal-regulating kinase 1, IAPs—inhibitors of apoptosis proteins, JAK—Janus kinase, STAT3—signal transducer and activator of transcription 3, mtDNA—mitochondrial DNA, GSH—glutathione, ROS—reactive oxygen species.
